# Association of triglyceride-glucose index and stroke recurrence among nondiabetic patients with acute ischemic stroke

**DOI:** 10.1186/s12883-022-02588-3

**Published:** 2022-03-08

**Authors:** Xiaomeng Yang, Guangyao Wang, Jing Jing, Anxin Wang, Xiaoli Zhang, Qian Jia, Xia Meng, Xingquan Zhao, Liping Liu, Hao Li, Yongjun Wang, Yilong Wang

**Affiliations:** 1 Department of Neurology, Beijing Tiantan Hospital, Capital Medical University, No.119, South 4th Ring West Road, Fengtai District, Beijing, 100070 China; 2 China National Clinical Research Center for Neurological Diseases, Beijing, China; 3 Department of Neurology, Beijing Jishuitan Hospital, Beijing, China; 4 Advanced Innovation Center for Human Brain Protection, Capital Medical University, Beijing, China

**Keywords:** Insulin resistance, Ischemic stroke, Prognosis, Triglyceride-glucose index, stroke recurrence, death

## Abstract

**Backgroud and purpose:**

Triglyceride-glucose (TyG) index has been considered a surrogate marker of insulin resistance. We investigated the association between TyG index and stroke recurrence and compared the effectiveness of TyG index with homeostasis model assessment of insulin resistance (HOMA-IR) in predicting stroke recurrence and death in nondiabetic acute ischemic stroke patients.

**Methods:**

Nondiabetic acute ischemic stroke patients from the ACROSS-China (Abnormal Glucose Regulation in Patients with Acute Stroke across China) registry were included. TyG index was performed and classified into four groups by quartiles. The outcomes were stroke recurrence and death within 1 year. The association between TyG index and the risk of stroke recurrence and death were analyzed by Cox regression models. Receiver operating characteristic (ROC) curve analysis was performed to evaluate the prediction of TyG index and HOMA-IR for stroke recurrence and death. Delong test was used for comparing the differences between area under the curve (AUC) of TyG index and HOMA-IR.

**Results:**

Among the 1226 patients included, the median (interquartile range) of TyG index was 5.8 (5.5–6.2). Both the third and fourth quartiles of TyG index were associated with an increased risk of stroke recurrence (adjusted hazard ratio 2.04, 95% confidence interval 1.26–3.31; adjusted hazard ratio 1.86, 95% confidence interval 1.13–3.06). Patients with fourth quartiles of TyG index were associated with a higher mortality risk (adjusted hazard ratio, 2.91; 95% confidence interval, 1.62–2.53). Regarding stroke recurrence within 1 year, the AUC (95% confidence interval) of the ROC curve for the TyG index was similar to that of the HOMA-IR[0.56 (0.52–0.61) vs 0.57 (0.52–0.61); *P* = 0.93]. Regarding death within 1 year, the AUCs (95% confidence interval) of the ROC curve for the TyG index and HOMA-IR were 0.55 (0.50–0.61) and 0.59 (0.53–0.64), respectively (*P* = 0.32).

**Conclusions:**

Elevated TyG index was associated with an increased risk of stroke recurrence and death. However, neither of TyG nor HOMA-IR can be a qualified predictor of stroke recurrence and death in nondiabetic acute ischemic stroke patients.

**Supplementary Information:**

The online version contains supplementary material available at 10.1186/s12883-022-02588-3.

## Introduction

Insulin resistance was common in subjects with multiple metabolic disorders and stroke [1, 2]. Insulin resistance, measured by homeostasis model assessment-insulin resistance (HOMA-IR) index, was associated with an increased risk of stroke recurrence in nondiabetic patients with acute ischemic stroke [3]. Previous studies have illustrated the importance of triglycerides in the pathogenesis of insulin resistance and the biological plausibility for using triglycerides levels as a surrogate in the identification of insulin resistance. Besides, the levels of fasting triglyceride and glucose are universally available in the clinical practice [4, 5]. Some studies showed that Triglyceride-glucose (TyG) index was more independently associated with the presence of coronary artery atherosclerosis [6], carotid atherosclerosis [7], and the development of diabetes [8] than HOMA-IR. Another study showed that TyG index was similar to HOMA-IR in prediction of diabetes [9]. However, few studies have investigated the relationship between TyG index and stroke recurrence or death and compared the effectiveness of TyG index and HOMA-IR in predicting stroke recurrence or death in nondiabetic acute ischemic stroke patients.

Using data from the ACROSS-China registry (Abnormal Glucose Regulation in Patients With Acute Stroke Across China), we aim to investigate the association between TyG index and stroke recurrence together with death, and compare effectiveness of TyG index and HOMA-IR in predicting stroke recurrence and death of nondiabetic acute ischemic stroke patients.

## Methods

### Study participants

The detailed design of the ACROSS-China study has  been described previously [10]. Briefly, ACROSS-China is a nationwide prospective cohort study which aimed to investigate the prevalence of abnormal glucose regulation in hospitalized patients with a first-ever ischemic and hemorrhagic stroke within 14 days after onset and the relationship of abnormal glucose regulation with the prognosis of stroke from 2008 to 2009 across China. The ACROSS-China study has been approved by the ethics committee of Beijing Tiantan Hospital, Capital Medical University and all participating hospitals. All participants or their legal representatives have provided written informed consent.

Acute ischemic stroke patients without a history of diabetes mellitus in the ACROSS-China study were included in this analysis. The definition of history of diabetes mellitus was based on the previous medical records and use of hypoglycemia agents. According to the World Health Organization criteria [11], acute ischemic stroke was diagnosed with confirmation by brain computed tomography or magnetic resonance imaging.

### Data collection

Patient information including demographic characteristics, medical history, and risk factors were recorded within 24 h after admission through face-to-face interviews by trained interviewers from the participating hospitals according to a standardized protocol. According to the TOAST (Trial of ORG 10,172 in Acute Stroke Treatment) criteria, the etiologic subtypes of acute ischemic stroke were classified [12]. The National Institutes of Health Stroke Scale (NIHSS) was used to assess the severity of neurological impairment within 24 h after admission. Complications of pulmonary or urinary infection and medications used during hospitalization were recorded.

### Evaluation of TyG index

The first overnight fasting venous blood samples (at least 8 h) were drawn to measure the fasting triglyceride. Triglyceride was enzymatically measured using spectrophotometric methods. Fasting venous blood samples were drawn to measure fasting glucose levels on day 14 ± 3 after stroke or before discharge (if length of stay in hospital was < 14 days) after overnight fasting (at least 8 h). Fasting glucose was measured by using an enzymatic method. TyG index was calculated as ln (fasting triglyceride [mg/dL] × fasting glucose [mg/dL]/2) [6].

### Evaluation of HOMA-IR

Fasting venous blood samples were drawn to measure fasting insulin on day 14 ± 3 after stroke or before discharge (if length of stay in hospital was < 14 days) after overnight fasting (at least 8 h). Fasting insulin was measured by using a competitive radioimmunoassay (Diagnostic Products Corporation). The fasting glucose used to calculate the HOMA-IR was the same as the TyG index. HOMA-IR was calculated as fasting insulin [μU/mL] × fasting glucose [mmol/L]/22.5) [13].

### Outcomes and follow-up

All patients enrolled in the ACROSS-China study were followed up at 12 months after stroke onset by a centralized telephone follow-up, which was based on a shared standardized interview protocol. The outcomes were stroke recurrence and death within 1 year. Stroke recurrence was defined as an aggravated primary neurological deficit, a new neurological deficit, or re-hospitalization caused by ischemic or hemorrhagic stroke [14].

### Statistical analysis

Patients were divided into four groups according to the quartiles of TyG index. Continuous variables were presented as median with interquartile range and categorical variables were presented as proportions. The baseline variables among different quartiles of TyG index were compared by Wilcoxon or Kruskal–Wallis test for continuous variables and χ2 test for categorical variables. The associations of TyG index with stroke recurrence and death were analyzed by using Cox proportional-hazards regression models. In the multivariable model, all the potential confounders listed in Table 1 were included. The area under the curve (AUC) of the receiver operating characteristics (ROC) curve and a 95% confidence interval were calculated to compare the predictive power of the HOMA-IR and TyG index for stroke recurrence and death. Delong test was used for comparing the differences between AUCs. We further evaluated the associations between TyG index and risk of outcomes in nondiabetic acute ischemic stroke patients without cardioembolism.

**Table 1:** Characteristics of patients included according to TyG index quartiles

Characteristics	Quartiles of the TyG index				*P* value
	Quartiles 1, 4.31–5.48	Quartiles 2, 5.48–5.81	Quartiles 3, 5.81–6.22	Quartiles 4, 6.22–8.17	
Patients, n (%)	306	307	307	306	
Male, n (%)	197(64.8)	182(59.5)	191(62.2)	202(66.7)	0.28
Age, y, median (IQR)	67.0(55.0–75.0)	65.0(53.0–75.0)	60.0(53.0–69.0)	59.0(51.0–70.0)	< 0.001
BMI, median (IQR)	24.0(21.6–26.2)	24.9(22.5–27.0)	24.9(22.9–27.3)	25.2(23.4–27.4)	< 0.001
Smoking, n (%)					0.49
Current smoker	99(32.4)	93(30.3)	99(32.2)	114(37.3)	
Ever smoker	31(10.1)	26(8.5)	34(11.1)	30(9.8)	
Nonsmoker	176(57.5)	188(61.2)	174(56.7)	162(52.9)	
Medical history					
Hypertension, n (%)	161 (52.6)	174(56.7)	198(64.5)	203(66.3)	0.001
Hyperlipidemia, n (%)	20(6.5)	28(9.1)	43(14.0)	50(16.3)	< 0.001
Atrial fibrillation, n (%)	34(11.1)	17(5.5)	14(4.6)	10(3.3)	< 0.001
Coronary heart disease, n (%)	32(10.5)	39(12.7)	39(12.7)	38(12.4)	0.80
Medication during hospitalization, n (%)					
Antihypertensive drugs	118(38.6)	136(44.3)	145(47.2)	144(47.1)	0.11
Statins	156(51.0)	138(45.0)	157(51.1)	176(57.5)	0.02
Intravenous alteplase	8(2.6)	7(2.3)	9(2.9)	12(3.9)	0.66
Antiplatelet	194(63.4)	187(60.9)	188(61.2)	194(63.4)	0.87
Anticoagulation	25(8.2)	22(7.2)	15(4.9)	18(5.9)	0.37
Complications during hospitalization, n (%)					
Pulmonary infection	29(9.5)	36(11.7)	21(6.8)	9(2.9)	< 0.001
Urinary infection	6(2.0)	18(5.9)	11(3.6)	7(2.3)	0.03
NIHSS at admission, median (IQR)	4(2–8)	5(3–9)	4(2–8)	4(2–6)	< 0.001
TOAST subtypes, n (%)					0.04
Large artery atherosclerosis	184(60.1)	194(63.2)	188(61.2)	185(60.5)	
Small artery occlusion	74(24.2)	72(23.5)	87(28.3)	90(29.4)	
Cardioembolism	30(9.8)	22(7.2)	18(5.9)	7(2.3)	
Other/undetermined	6(2.0)	9(2.9)	5(1.6)	12(3.9)	
Undefined	12(3.9)	10(3.3)	9(2.9)	12(3.9)	

*Abbreviations:* TyG index indicates triglyceride glucose index, *BMI* Body mass index, *IQR* interquartile range, *NIHSS* National Institutes of Health Stroke Scale, *TOAST* Trial of Org 10,172 in Acute Stroke Treatment

## Results

### Study participants

**Fig. 1:**
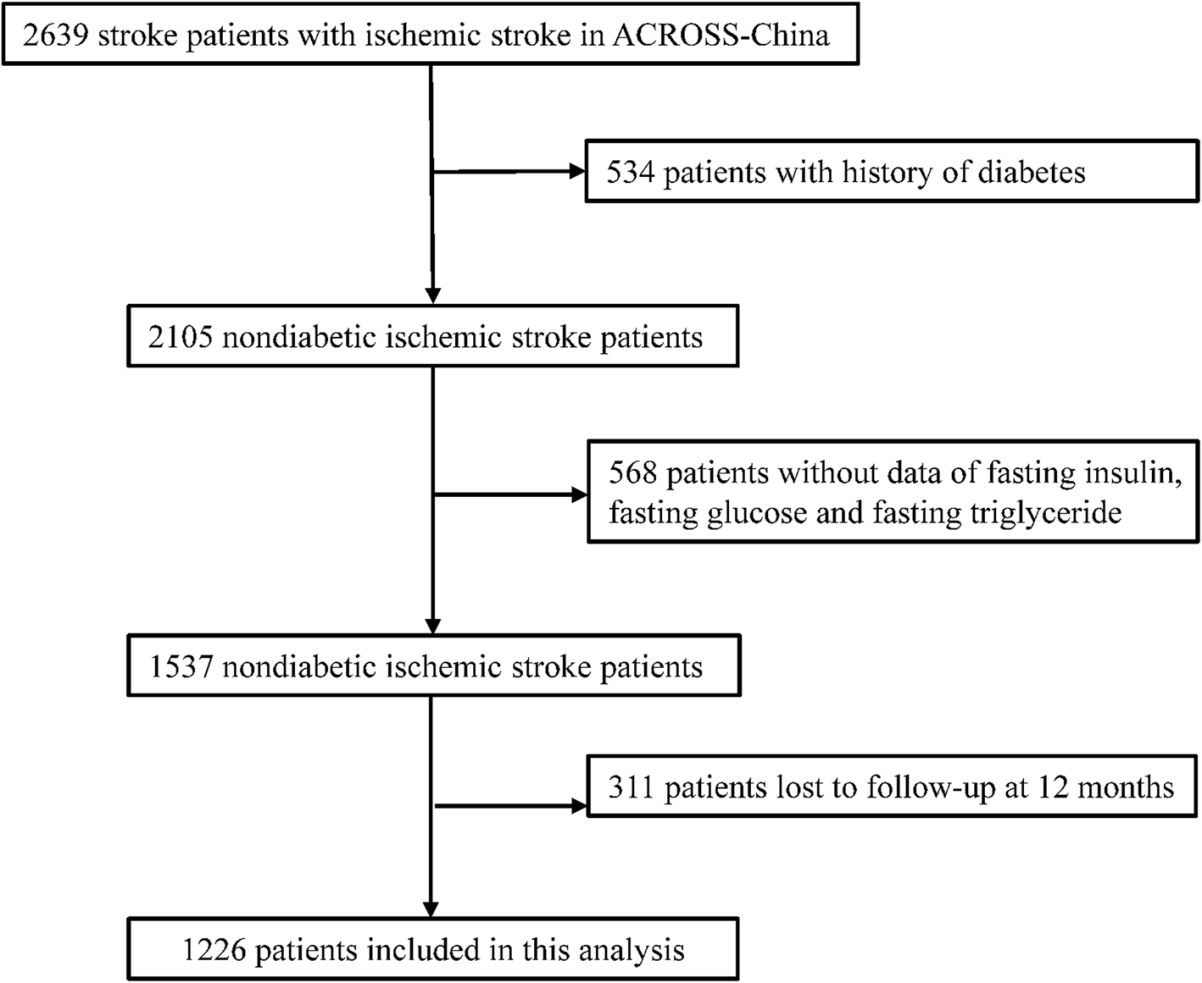
Flow chart of patient selection. Abbreviations: ACROSS- China indicates Abnormal Glucose Regulation in Patients With Acute Stroke Across China

A total of 2639 patients with acute ischemic stroke were enrolled in the ACROSS-China study. After excluding 534 patients with a history of diabetes mellitus, 568 patients with missing data on fasting insulin, fasting glucose and fasting triglyceride, 311 patients lost to follow up at 12-months, 1226 nondiabetic acute ischemic stroke patients were included in this analysis (Fig. 1). Baseline characteristics of nondiabetic acute ischemic stroke patients included and not included were almost balanced, except that the patients included in this analysis were more likely to have higher body mass index and higher proportion of taking anticoagulation during hospitalization (Supplemental Table 1).

### Baseline characteristics

The baseline characteristics of nondiabetic acute ischemic stroke patients according to quartiles of TyG index are shown in Table 1. The median age was 62 years, 63.3% of them were male, and the median of TyG index was 5.8. Patients with higher TyG index were more likely to be younger; have slightly higher body mass index; have higher proportions of history of hypertension and hyperlipidemia; have lower proportions of history of atrial fibrillation; take statins during hospitalization; and have lower NIHSS at admission.

### Clinical outcomes according to TyG index and HOMA-IR index

Table 2 shows the 1-year outcomes after nondiabetic acute ischemic stroke across the quartiles of TyG index. The cumulative occurrence of stroke recurrence and death was 14.5% and 9.3% at 1-year follow up. Patients with higher TyG index quartile showed a higher incidence of stroke recurrence within 1 year (log-rank test *P* = 0.03). After adjusted for age, sex, body mass index, smoking status, medical history of hypertension, hyperlipidemia, atrial fibrillation and coronary heart disease, antihypertensive drugs, statins, intravenous alteplase, antiplatelet and anticoagulation during hospitalization, pulmonary infection and urinary infection during hospitalization, NIHSS at admission and Trial of Org 10 172 in acute stroke treatment (TOAST) subtypes, patients in the third and fourth quartile of TyG index were associated with an increased risk of stroke recurrence (adjusted hazard ratio 2.04, 95% confidence interval 1.26–3.31; adjusted hazard ratio 1.86, 95% confidence interval 1.13–3.06). Patients with fourth quartiles of TyG index were associated with a higher mortality risk (adjusted hazard ratio, 2.91; 95% confidence interval, 1.62–2.53; *P* < 0.001).Similar results were observed in nondiabetic acute ischemic stroke patients without cardioembolism (Supplemental Table 2 and 3).

**Table 2:** Adjusted hazard ratio of outcomes within 1 year according to TyG index quartiles

Prognosis	TyG index	n	Events, n (%)	Model 1	*P* Value	Model 2^a^	*P* Value
				Unadjusted HR (95% CI)		Adjusted HR (95% CI)	
Stroke recurrence	Q1 (4.31–5.48)	298	31(10.4)	Reference		Reference	
	Q2 (5.48–5.81)	290	37(12.8)	1.38(0.83–2.29)	0.21	1.33(0.80–2.23)	0.27
	Q3 (5.81–6.22)	301	53(17.6)	1.97(1.23–3.16)	0.005	2.04(1.26–3.31)	0.004
	Q4 (6.22–8.17)	296	51(17.2)	1.86(1.15–3.00)	0.01	1.86(1.13–3.06)	0.01
	*P* for trend				0.03		0.03
Death	Q1 (4.31–5.48)	306	19(6.2)	Reference		Reference	
	Q2 (5.48–5.81)	307	34(11.1)	1.85(1.05–3.24)	0.03	1.94(1.09–3.48)	0.03
	Q3 (5.81–6.22)	307	23(7.5)	1.14(0.62–2.10)	0.69	1.56(0.83–2.93)	0.17
	Q4 (6.22–8.17)	306	38(12.4)	2.05(1.18–3.56)	0.01	2.91(1.62–5.23)	< 0.001
	*P* for trend				0.045		0.002

*Abbreviations:* TyG index indicates triglyceride glucose index, *HR* Hazard ratio

^a^Adjusted for age, sex, body mass index, smoking status, medical history of hypertension, hyperlipidemia, atrial fibrillation and coronary heart disease, antihypertensive drugs, statins, intravenous alteplase, antiplatelet and anticoagulation during hospitalization, pulmonary infection and urinary infection during hospitalization, NIHSS at admission and TOAST subtypes

**Fig. 2:**
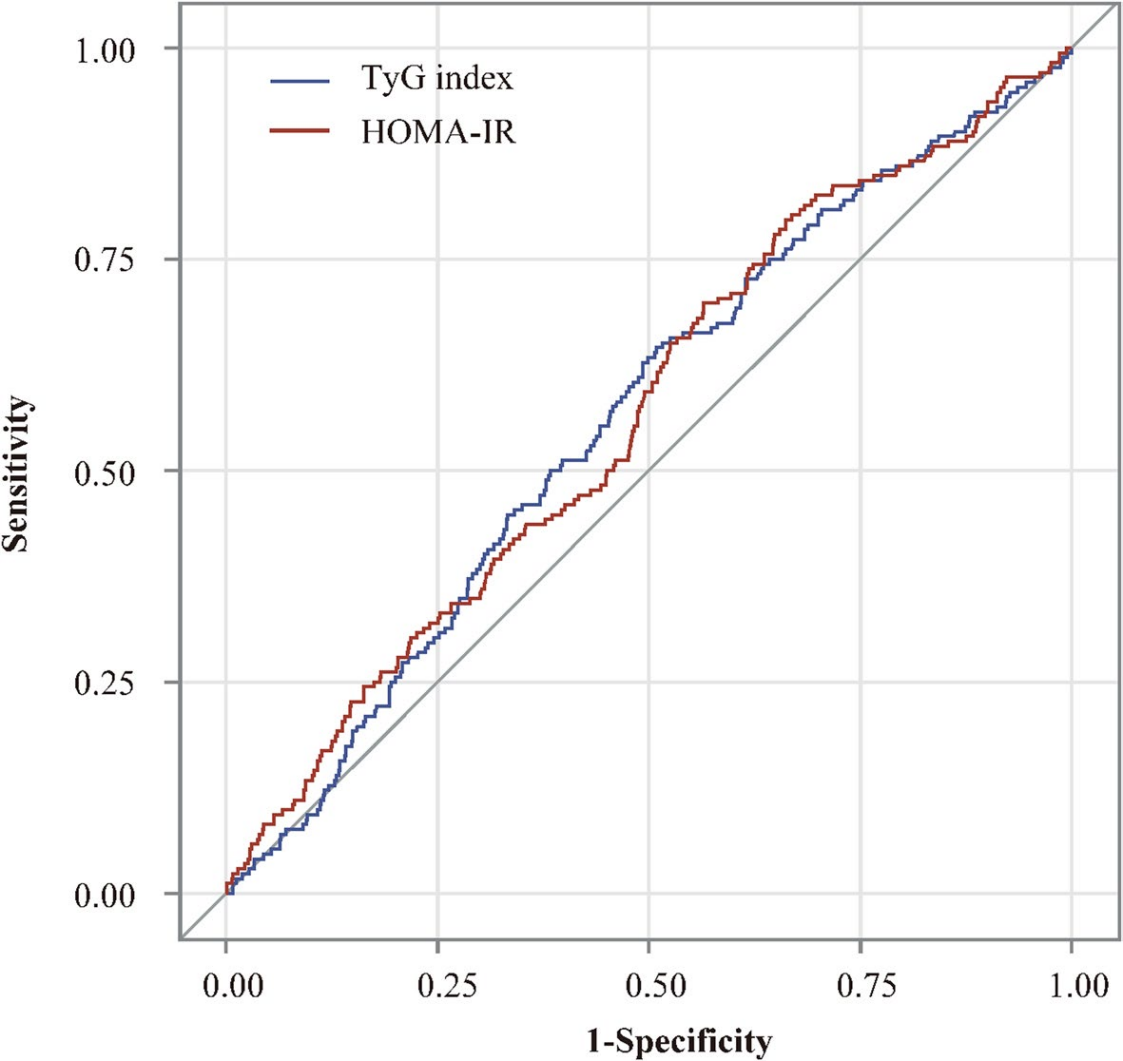
Receiver operating characteristic curves of TyG index and HOMA-IR for predicting stroke recurrence within 1 year. Abbreviations: TyG index indicates triglyceride-glucose index; HOMA-IR, homeostasis model assessment of insulin resistance

**Fig. 3:**
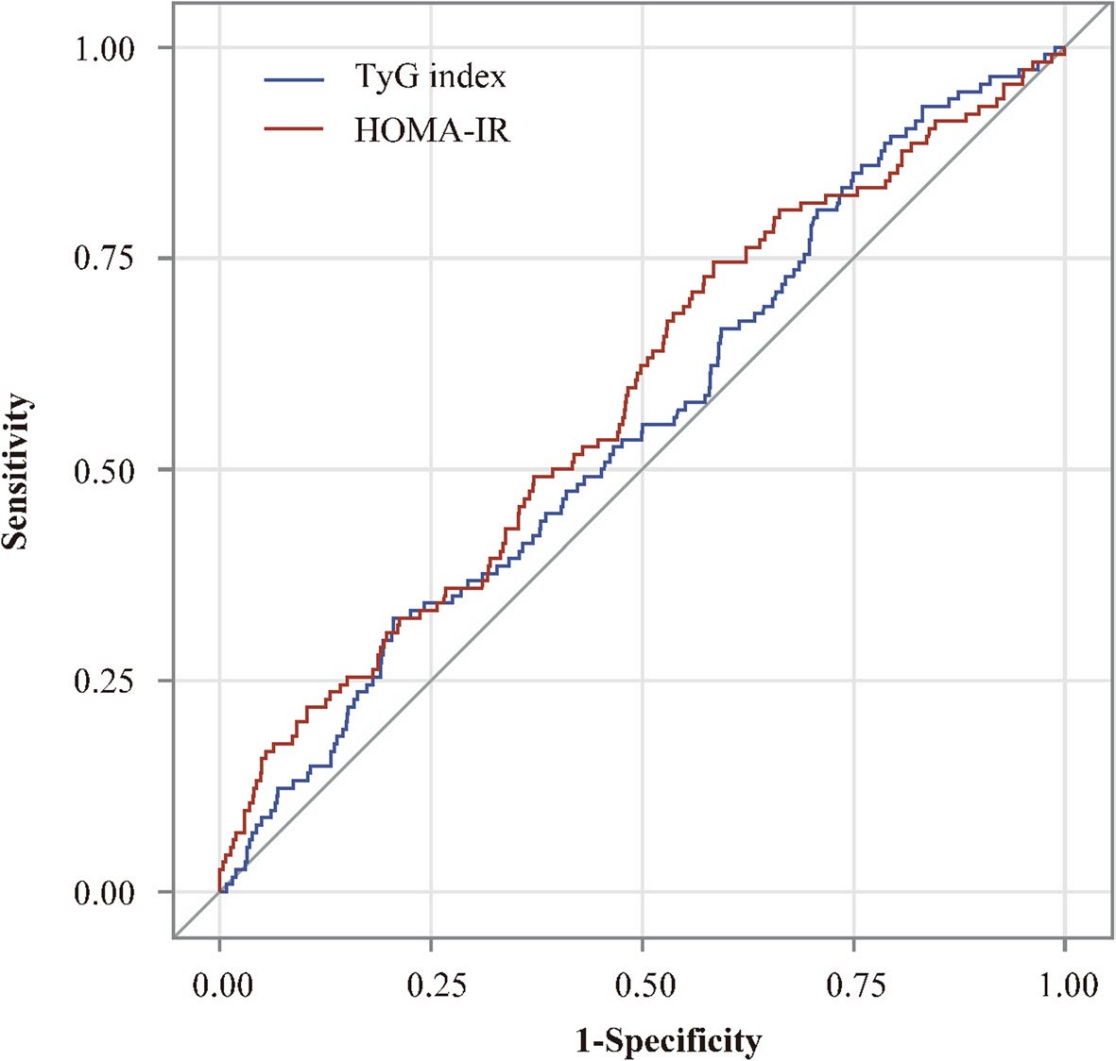
Receiver operating characteristic curves of TyG index and HOMA-IR for predicting death within 1 year. Abbreviations: TyG index indicates triglyceride-glucose index; HOMA-IR, homeostasis model assessment of insulin resistance

To further evaluate the predictive values of TyG index and HOMA-IR in predicting stroke recurrence and death in patients with nondiabetic acute ischemic stroke, the ROC curves and AUC regarding stroke recurrence and death were created (Fig. 2 and Fig. 3). Regarding stroke recurrence within 1 year, the AUCs (95% confidence interval) of the ROC curve for the TyG index and HOMA-IR were 0.56 (0.52–0.61) and 0.57 (0.52–0.61), respectively (*P* = 0.93). Regarding death within 1 year, the AUCs (95% confidence interval) of the ROC curve for the TyG index and HOMA-IR were  0.55 (0.50–0.61) and 0.59 (0.53–0.64), respectively (*P* = 0.32) (Fig. 3).

## Discussion

In the current study, we found neither of TyG nor HOMA-IR can be a qualified predictor of stroke recurrence and death in nondiabetic acute ischemic stroke patients. Although patients with higher TyG index quartile showed a higher risk of stroke recurrence and death within 1 year, from the viewpoint of clinical relevance, the curves in ROC analysis are drawn close to the diagonal line and area under curves of both TyG and HOMA-IR are relatively small (0.56 and 0.57), and thus their values of predicting stroke recurrence and death are judged to be low.

The rate of stroke recurrence within 1 year was 14.5%, which was higher than that in other countries [15] and similar to that in China [16, 17]. China National Stroke Registry (CNSR) study conducted between September 2007 and August 2008 showed the rate of stroke recurrence within 1 year was 19.3% [17]. Nanjing Stroke Registry demonstrated that the stroke recurrence rate within 1 year was 11.2% [16]. Several reasons might explain for the higher risk of stroke recurrence in China. Firstly, the prescription of secondary preventive medications at discharge and persistence of secondary preventive medications after stroke in China were lower than other countries [18–21]. Secondly, insulin resistance was associated with atherosclerosis [22, 23]. Intracranial artery stenoses was found commonly among stroke patients in Asian [24–26]. Intracranial stenosis or occlusion after acute cerebral ischemia was associated with higher risk of stroke recurrence [26]. The rate of stroke recurrence was 14.5% in this study, which probably reflected a high proportion of intracranial stenosis.

Glucose clamp technique is the gold standard test for quantifying insulin resistance [27], but it is limited to research purposes because of being complicated, time-consuming and expensive. HOMA-IR based on insulin and glucose was developed as a convenient method for measuring insulin resistance, which reflected insulin resistance mainly in the liver [28]. The TyG index based on the triglyceride and glucose was even simpler to calculate, which reflected insulin resistance mainly in the skeletal muscle [29, 30].

Previous studies showed that TyG index was better associated with carotid atherosclerosis [7], nonalcoholic fatty liver disease [31], diabetes [8] and coronary artery atherosclerosis [6] than HOMA-IR. Previous studies also demonstrated that compared with the HOMA-IR, TyG index had higher sensitivity and lower specificity for recognizing insulin resistance among apparently healthy subjects [4]. However, our study showed that the predictive value of TyG index and HOMA-IR was similar in predicting stroke recurrence within 1 year. Caution is still required in our interpretations because fasting triglyceride and glucose were not measured at the same day. Further studies are needed to compare the predictive value of TyG index and HOMA-IR on stroke recurrence after nondiabetic acute ischemic stroke.

Although the mechanism underlying the association between the TyG index and stroke recurrence has not been clarified clearly, which may be related to insulin resistance. Insulin resistance was a syndrome that was associated with a clustering of metabolic disorders [32]. Insulin resistance was associated with obesity, diabetes mellitus, hypertension, hyperlipemia, inflammation, and atherosclerosis [32, 33],which were important risk factors of stroke recurrence [34]. Additionally, our previous studies showed insulin resistance measured by post-glucose load measures was associated with stroke recurrence [35], which was in line with TyG index in this analysis. Several potential mechanisms might account for the association between TyG index and insulin resistance. First, insulin resistance was associated with higher levels of triglyceride [36]. In return, serum levels of triglyceride was associated with insulin action in non-insulin-dependent diabetes mellitus populations [37]. Experiments showed that relative inability to store triglyceride in the subcutaneous depot might indicate a mechanism contributing to insulin resistance in the setting of obesity [38]. Second, hyperglycemia might impair insulin sensitivity [39].

There were several limitations in this study. First, among the 2105 nondiabetic acute ischemic stroke patients, 920 (43.7%) were excluded because they were not tested for fasting glucose, triglycerides or insulin or lost to follow-up at 1 year. However, the baseline characteristics between patients included and those excluded were almost balanced. Second, compared with acute ischemic stroke patients in western countries, those in China had a higher incidence of large artery atherosclerosis. This indicated that our findings may not apply to other populations of patients with acute ischemic stroke. Third, selection bias may have existed because most of the patients was mild ischemic stroke in our study. As a standard oral glucose tolerance test was required in all the patients without prior diabetes mellitus in the ACROSS-China registry, several patients with severe clinical symptom could not complete the oral glucose tolerance test and were excluded. Fourth, the fasting triglyceride used to calculate TyG index was measured by first overnight fasting venous blood samples. The fasting insulin and fasting glucose were measured on 14 ± 3 days after stroke or before discharge. However, fasting glucose measured on 14 ± 3 days after stroke or before discharge may reduce the proportion of stress hyperglycemia [40]. Further studies about comparing the predictive value between TyG index and gold standard methods for insulin resistance are needed to confirm the results in other populations.

In conclusions, neither of TyG nor HOMA-IR can be a qualified predictor of stroke recurrence in nondiabetic acute ischemic stroke patients.

## Supplementary Information

**Additional file 1:** **Table1. **Baseline characteristics of nondiabetic acute ischemic stroke patients included versus not included. **Table 2.** Characteristics of nondiabetic acute ischemic stroke patients without cardioembolism according to TyG index quartiles. **Table 3.** Adjusted hazard ratio of outcomes within 1 year according to TyG index quartiles in nondiabetic acute ischemic stroke patients without cardioembolism.

## Data Availability

The datasets during and/or analysed during the current study available from the corresponding author on reasonable request.
