# Supplementary material for: Association of triglyceride-glucose index and stroke recurrence among nondiabetic patients with acute ischemic stroke
Source: BMC Neurol. 2022 Mar 8;22:79. doi: 10.1186/s12883-022-02588-3 (PMC8902785; doi:10.1186/s12883-022-02588-3)
Supplement: Supplementary file 1 — Additional file 1: Table1. Baseline characteristics of nondiabetic acute ischemic stroke patients included versus not included. Table 2. Characteristics of nondiabetic acute ischemic stroke patients without cardioembolism according to TyG index quartiles. Table 3. Adjusted hazard ratio of outcomes within 1 year according to TyG index quartiles in nondiabetic acute ischemic stroke patients without cardioembolism. [file 12883_2022_2588_MOESM1_ESM.docx]

**Supplemental Material**

**Supplemental tables**

**Table 1** Baseline characteristics of nondiabetic acute ischemic stroke patients included versus not included

| Characteristics | Included | Not included | *P* value |  |
| --- | --- | --- | --- | --- |
|  |  |  |  |  |
| Patients, n (%) | 1226 | 879 |  |  |
| Male, n (%) | 772(63.3) | 585(66.9) | 0.09 |  |
| Age, y, median (IQR) | 62.0(53.0-73.0) | 63.0(53.0-73.0) | 0.57 |  |
| BMI, median (IQR) | 24.8(22.6-27.0) | 24.2(22.2-26.2) | <0.001 |  |
| Smoking, n (%) |  |  | 0.93 |  |
| Current smoker | 405(33.0) | 286(32.5) |  |  |
| Ever smoker | 121(9.9) | 84(9.6) |  |  |
| Nonsmoker | 700(57.1) | 509(57.9) |  |  |
| Medical history |  |  |  |  |
| Hypertension, n (%) | 736(60.0) | 527(60.0) | 1.00 |  |
| Hyperlipidemia, n (%) | 141(11.5) | 83(9.4) | 0.13 |  |
| Atrial fibrillation, n (%) | 75(6.1) | 60(6.8) | 0.53 |  |
| Coronary heart disease, n (%) | 148(12.1) | 111(12.6) | 0.74 |  |
| Medication during hospitalization, n (%) |  |  |  |  |
| Antihypertensive drugs | 543(44.3) | 357(40.6) | 0.10 |  |
| Statins | 627(51.1) | 423(48.1) | 0.18 |  |
| Intravenous alteplase | 36(2.9) | 28(3.2) | 0.80 |  |
| Antiplatelet | 763(62.2) | 557(63.4) | 0.62 |  |
| Anticoagulation | 80(6.5) | 38(4.3) | 0.03 |  |
| Complications during hospitalization, n (%) |  |  |  |  |
| Pulmonary infection | 95(7.7) | 67(7.6) | 0.93 |  |
| Urinary infection | 42(3.4) | 32(3.6) | 0.81 |  |
| NIHSS at admission, median (IQR) | 4(2-8) | 4(2-8) | 0.10 |  |
| TOAST subtypes, n (%) |  |  | 0.22 |  |
| Large artery atherosclerosis | 751(61.3) | 562(63.9) |  |  |
| Small artery occlusion | 323(26.3) | 194(22.1) |  |  |
| Cardioembolism | 77(6.3) | 59(6.7) |  |  |
| Other/undetermined | 32(2.6) | 25(2.8) |  |  |
| Undefined | 43(3.5) | 39(4.4) |  |  |

Abbreviations: TyG index indicates triglyceride glucose index; BMI, body mass index; IQR, interquartile range; NIHSS, National Institutes of Health Stroke Scale; TOAST, Trial of Org 10172 in Acute Stroke Treatment.

**Table 2** Characteristics of nondiabetic acute ischemic stroke patients without cardioembolism according to TyG index quartiles

| Characteristics | Quartiles of the TyG index | | | | *P* value |
| --- | --- | --- | --- | --- | --- |
|  | Quartiles 1,  4.31-5.49 | Quartiles 2,  5.49-5.83 | Quartiles 3,  5.83-6.24 | Quartiles 4,  6.25-8.17 |  |
| Patients, n (%) | 287 | 287 | 288 | 287 |  |
| Male, n (%) | 196(68.8) | 175(61.2) | 186(64.6) | 190(66.9) | 0.26 |
| Age, y, median (IQR) | 67.0(55.0-75.0) | 64.0(53.0-74.0) | 59.0(52.0-68.0) | 59.0(51.0-70.0) | <0.001 |
| BMI, median (IQR) | 24.2(21.7-26.2) | 25.0(22.6-27.0) | 25.4(23.3-27.7) | 25.2(23.2-27.4) | <0.001 |
| Smoking, n (%) |  |  |  |  | 0.57 |
| Current smoker | 98(34.1) | 91(31.7) | 95(33.0) | 108(37.6) |  |
| Ever smoker | 29(10.1) | 24(8.4) | 34(11.8) | 28(9.8) |  |
| Nonsmoker | 160(55.7) | 172(59.9) | 159(55.2) | 151(52.6) |  |
| Medical history |  |  |  |  |  |
| Hypertension, n (%) | 154 (53.7) | 166(57.8) | 192(66.7) | 189(65.9) | 0.002 |
| Hyperlipidemia, n (%) | 20(7.0) | 27(9.4) | 44(15.3) | 46(16.0) | 0.001 |
| Atrial fibrillation, n (%) | 12(4.2) | 4(1.4) | 6(2.1) | 4(1.4) | 0.08 |
| Coronary heart disease, n (%) | 24(8.4) | 31(10.8) | 33(11.5) | 35(12.2) | 0.48 |
| Medication during hospitalization, n (%) |  |  |  |  |  |
| Antihypertensive drugs | 110(38.3) | 123(42.9) | 141(49.0) | 133(46.3) | 0.06 |
| Statins | 148(51.6) | 132(46.0) | 154(53.5) | 162(56.4) | 0.08 |
| Intravenous alteplase | 7(2.4) | 8(2.8) | 8(2.8) | 12(4.2) | 0.63 |
| Antiplatelet | 186(64.8) | 180(62.7) | 177(61.5) | 182(63.4) | 0.87 |
| Anticoagulation | 15(5.2) | 19(6.6) | 12(4.2) | 16(5.6) | 0.63 |
| Complications during hospitalization, n (%) |  |  |  |  |  |
| Pulmonary infection | 25(8.7) | 27(9.4) | 14(4.9) | 9(3.1) | 0.005 |
| Urinary infection | 4(1.4) | 14(4.9) | 10(3.5) | 6(2.1) | 0.07 |
| NIHSS at admission, median (IQR) | 4(2-8) | 5(2-9) | 4(2-7) | 4(2-6) | 0.001 |
| TOAST subtypes, n (%) |  |  |  |  | 0.86 |
| Large artery atherosclerosis | 193(67.2) | 194(67.6) | 187(64.9) | 177(61.7) |  |
| Small artery occlusion | 75(26.1) | 75(26.1) | 85(29.5) | 88(30.7) |  |
| Other/undetermined | 7(2.4) | 8(2.8) | 6(2.1) | 11(3.8) |  |
| Undefined | 12(4.2) | 10(3.5) | 10(3.5) | 11(3.8) |  |

Abbreviations: TyG index indicates triglyceride glucose index; BMI, body mass index; IQR, interquartile range; NIHSS, National Institutes of Health Stroke Scale; TOAST, Trial of Org 10172 in Acute Stroke Treatment.

**Table 3** Adjusted hazard ratio of outcomes within 1 year according to TyG index quartiles in nondiabetic acute ischemic stroke patients without cardioembolism

| Prognosis | TyG index | n | Events, n (%) | Model 1 | *P* Value | Model 2* | *P* Value |
| --- | --- | --- | --- | --- | --- | --- | --- |
|  |  |  |  | Unadjusted HR (95% CI) |  | Adjusted HR (95% CI) |  |
| Stroke recurrence | Q1 (4.31-5.49) | 283 | 30(10.6) | Reference |  | Reference |  |
|  | Q2 (5.49-5.83) | 274 | 34(12.4) | 1.32(0.78-2.23) | 0.30 | 1.27(0.75-2.15) | 0.38 |
|  | Q3 (5.83-6.24) | 285 | 52(18.2) | 2.03(1.25-3.29) | 0.004 | 2.12(1.29-3.46) | 0.003 |
|  | Q4 (6.25-8.17) | 277 | 48(17.3) | 1.84(1.13-3.00) | 0.02 | 1.84(1.11-3.04) | 0.02 |
|  | *P* for trend |  |  |  | 0.02 |  | 0.03 |
| Death | Q1 (4.31-5.49) | 287 | 15(5.2) | Reference |  | Reference |  |
|  | Q2 (5.49-5.83) | 287 | 28(9.8) | 1.92(1.03-3.60) | 0.04 | 1.95(1.03-3.70) | 0.04 |
|  | Q3 (5.83-6.24) | 288 | 20(6.9) | 1.24(0.63-2.45) | 0.53 | 1.61(0.81-3.22) | 0.18 |
|  | Q4 (6.25-8.17) | 287 | 38(13.2) | 2.64(1.45-4.80) | 0.002 | 3.20(1.72-5.96) | <0.001 |
|  | *P* for trend |  |  |  | 0.01 |  | 0.003 |

Abbreviations: TyG index indicates triglyceride glucose index; HR, hazard ratio.

*Adjusted for age, sex, body mass index, smoking status, medical history of hypertension, hyperlipidemia, atrial fibrillation and coronary heart disease, antihypertensive drugs, statins, intravenous alteplase, antiplatelet and anticoagulation during hospitalization, pulmonary infection and urinary infection during hospitalization, NIHSS at admission and TOAST subtypes.
